# Intravascular lipoma of the renal vein

**DOI:** 10.1259/bjrcr.20150072

**Published:** 2015-06-10

**Authors:** Z Doyle, B Wolford, M M Morshedi, C S Santillan

**Affiliations:** ^1^School of Medicine, University of California San Diego, La Jolla, CA, USA; ^2^Department of Radiology, University of California San Diego, San Diego, CA, USA; ^3^Department of Radiology, University of California Los Angeles, Los Angeles, CA, USA

## Abstract

Lipomas are benign neoplasms composed of adipocytes encased in a fibrous capsule. Intravascular lipomas are rare and almost always incidental findings. In the published literature, the majority are described within the inferior vena cava (IVC) and less frequently reported in the superior vena cava, brachiocephalic vein, subclavian vein, internal jugular vein, external iliac vein and common femoral vein. We present the case of a 59-year-old male who presented with a symptomatic ureteral calculus and was found to have an intravascular lipoma of the right renal vein with extension into the IVC. To our knowledge, this is the first ever report of an intravascular lipoma in the renal vein. We discuss the imaging characteristics of intravascular lipomas and the differential diagnosis that should be considered.

## Clinical Presentation

A 59-year-old male initially presented to an outside hospital with right flank pain and gross haematuria due to a partially obstructing right ureteral calculus.

## Imaging Findings

**Figure 1:**
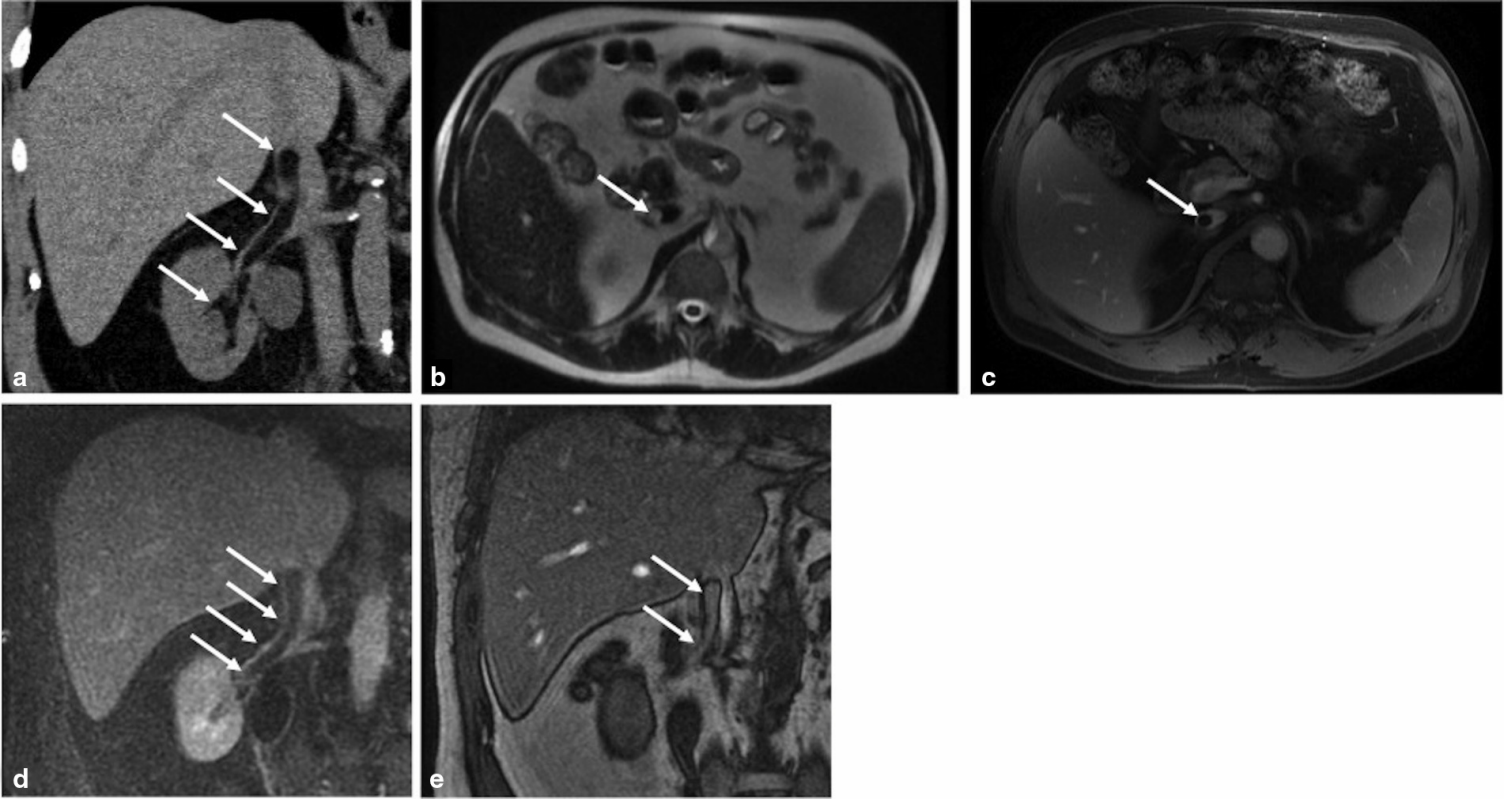
. Coronal CT image (a) demonstrates a well-circumscribed homogeneous fat-attenuating intravascular mass (arrows) extending from the renal veins to the inferior vena cava (IVC). Axial *T*_2_ MR image (b) shows a hyperintense round mass in the IVC that subtracts out on post-contrast *T*_1_ fat-saturated axial (c) and coronal (d) MR images as well as demonstrates chemical shift artefact on out-of-phase coronal MRI (e). The lesion is space filling, non-enhancing and nearly all fat, most compatible with an intravascular lipoma.

A non-contrast CT scan of the abdomen/pelvis performed at the time of diagnosis incidentally revealed a homogeneous smooth tubular fat-attenuating mass within the anterior right renal vein, extending into the inferior vena cava (IVC) and terminating at the level of the proximal intrahepatic portion of the vena cava (Figure 1a). The patient was referred to our institution for an MRI to further characterize the mass. The MRI showed that the well-circumscribed mass was hyperintense on T2 imaging, subtracted out on fat-saturated images, showed chemical shift artefact on out-of-phase imaging and did not enhance with contrast (Figure 1b–e). These findings were compatible with a lipoma extending from the segmental branches of the right renal vein to the main renal vein and into the IVC. The mass occupied the majority of the lumen of the main renal vein and approximately 50% of the lumen of the affected IVC.

A review of the currently available literature on PubMed was performed by using the search terms “lipoma”, “intravascular lipoma”, “intravenous lipoma”, “renal vein lipoma”, “inferior vena cava lipoma”, “IVC lipoma” and “renal lipoma” that found 27 reported cases of intravascular lipoma. Imaging characteristics are typical for a lipoma, with nearly all imaging modalities demonstrating a homogeneous, circumscribed, non-enhancing fatty mass. CT imaging typically shows a well-defined, ovoid, non-enhancing, hypoattenuating mass consistent with fat density. Intravenous contrast demonstrates a filling defect, corresponding to the intraluminal location of the lipomas, although some published cases report both intra- and extravascular extension.^1,2^ MRI was obtained in eight published case reports^2-9^ to confirm location and fatty composition, and demonstrated non-enhancing, intravascular space occupying, *T*_1_ and *T*2 hyperintense, circumscribed fatty lesions that subtract out on fat-subtraction imaging, similar to our case described above. Others have used angiography to assess the level of obstruction, and in one angiographic study, the obstruction was demonstrated and abnormal venous collaterals were seen, suggesting longstanding disruption of venous flow.^2^ However, angiography is not routinely performed if other imaging modalities do not show significant vascular obstruction.

## Differential Diagnosis

In addition to lipomas, the differential diagnosis of fat-containing intravascular masses includes a number of benign and malignant disease processes that may be differentiated by imaging. In the case of a fatty mass, liposarcoma must be considered. Typically, on imaging, liposarcomas appear as heterogeneous lesions. In contrast, lipomas are characteristically composed entirely of adipose tissue and therefore demonstrate homogeneous fatty attenuation. However, one will note that 31% of lipomas have non-adipose areas on imaging, a feature that is more typical of liposarcomas.^10^ Other features that suggest malignancy include thick septa, nodular and/or globular areas of non-adipose tissue, associated non-adipose masses and total non-adipose tissue comprising more than 25% of the lesion.^10^ Gaskin and Helms found that MRI was 100% specific in diagnosing a simple lipoma when a fatty mass contained no or only a few thin septa and minimal or no areas of enhancement or high *T*_2_ signal.^11^

Another differential diagnosis is renal angiomyolipoma. Angiomyolipomas are nearly always heterogeneous tumours composed of fat and soft-tissue material, unlike lipomas, which are primarily fat. Although there have been reported cases of renal angiomyolipomas involving the renal vein or IVC,^12^ angiomyolipomas arise from the renal parenchyma and present with a renal parenchymal defect with a direct juncture between normal renal parenchyma and the tumour.^13^

A renal cell carcinoma (RCC) with intravascular extension is another consideration. RCCs are almost always solid, enhancing exophytic masses. Their appearance is variable, as they may contain low-density cystic areas. Small tumours may be well marginated, but larger tumours—the ones that are typically associated with intravascular extension—have less of a distinct interface with the renal parenchyma.^14^ There are rare cases in the published literature of RCCs containing small amounts of fat.^15,16^ However, the amount of fat is minimal and these tumours usually have associated calcifications, with only a few reports of fat-containing RCCs without calcifications.^16,17^

Rarely, retroperitoneal leiomyosarcomas may be found in a completely intravascular pattern, with several published reports of leiomyosarcomas arising from the renal vein and the IVC.^18,19^ They are typically seen on CT and MRI as non-fatty, lobulated soft-tissue masses of intermediate attenuation signal, with areas of low attenuation that correspond to necrosis.^19,20^

Haemangioendothelioma refers to a heterogeneous group of low-grade vascular neoplasms, which display numerous histopathological characteristics.^21^ On imaging, these lesions resemble non-specific soft-tissue masses and may be associated with calcifications, oedema or haemorrhage.^22^ They invariably show some degree of enhancement, but are not intrinsically fat-containing.^22^ First described by Masson in 1923,^23^ intravascular haemangioendothelioma is a lesion characterized by endothelial proliferation within medium-sized veins; however, only rare cases have been reported in the abdomen.^24^

## Treatment

Management of intravascular lipoma varies in the literature. Because fat-specific sequences in MRI are able to characterize lesions with high specificity and differentiate fatty tissue from solid components, biopsy has typically been seen as unnecessary when imaging demonstrates a lesion composed entirely of fat and the patient is asymptomatic. However, lesions with large lipomatous components may be more difficult to accurately diagnose on imaging, such as benign lipomas *vs* well-differentiated liposarcomas.^25^ Biopsy of such lesions would be reasonable, although diagnostic biopsy was not performed in any of the reported cases of intravascular lipomas; cases that elected to obtain histological confirmation did so by complete excision of the lesion instead. It should also be noted that biopsy of heterogeneous lesions also has its own problems of sampling error and may lead to a false-negative result.^26^

Often, surgical resection is advised only if patients are symptomatic. Intravascular lipomas are most often asymptomatic, but can rarely cause venous obstructive symptoms, such as superior vena cava syndrome. Bravi et al^2^ describe a patient who developed a thrombotic complication owing to the occlusive effects of a lipoma preventing adequate venous return. In 2 of the 27 reported cases that were reviewed, the primary team elected to do surgery despite a lack of symptoms in order to prevent potential obstructive and thromboembolic complications, and to rule out malignant disease by providing a definitive histological diagnosis.^7,27^ Although an intravenous lesion may alter flow dynamics, there was no evidence of collateral formation or venous thrombosis to suggest a clinically significant flow obstruction in this case. The pedicle of implantation in this patient was not clear and therefore embolization could be a potential concern, but given that these lesions are well-encapsulated and likely arise from the vascular wall, embolization was thought to be unlikely to occur. For these reasons and because there was no evidence of malignant potential on imaging, the primary team elected for conservative management through surveillance. A repeat CT scan at 4 months showed no change in size or character of the lesion and no evidence of vascular obstruction or thrombosis. The patient remained asymptomatic on follow-up.

## Learning Points

1. On imaging, an intravascular lipoma is seen as a well-defined, homogeneous, non-enhancing, fat-attenuating mass without areas of soft tissue, haemorrhage or necrosis. A hyperintense signal on *T*_2_ imaging that subtracts on fat-saturated images confirms the diagnosis.
2. Intravascular lipomas must be distinguished from their more malignant counterpart— liposarcomas—given that a subset of lipomas can have soft-tissue components and may be indistinguishable on imaging alone.
3. Although benign, intravascular lipomas may require surgery depending on concern for obstructive complications.
